# Fixel-Based Analysis Effectively Identifies White Matter Tract Degeneration in Huntington’s Disease

**DOI:** 10.3389/fnins.2021.711651

**Published:** 2021-09-13

**Authors:** Sher Li Oh, Chiung-Mei Chen, Yih-Ru Wu, Maria Valdes Hernandez, Chih-Chien Tsai, Jur-Shan Cheng, Yao-Liang Chen, Yi-Ming Wu, Yu-Chun Lin, Jiun-Jie Wang

**Affiliations:** ^1^Lee Kong Chian School of Medicine, Nanyang Technological University, Singapore, Singapore; ^2^Department of Neurology, Chang Gung Memorial Hospital, Linkou, Taoyuan, Taiwan; ^3^College of Medicine, Chang Gung University, Taoyuan, Taiwan; ^4^Row Fogo Centre for Research into Ageing and the Brain, Department of Neuroimaging Sciences, The University of Edinburgh, Edinburgh, United Kingdom; ^5^Centre for Clinical Brain Sciences, The University of Edinburgh, Edinburgh, United Kingdom; ^6^Healthy Aging Research Center, Chang Gung University, Taoyuan, Taiwan; ^7^Clinical Informatics and Medical Statistics Research Center, College of Medicine, Chang Gung University, Taoyuan, Taiwan; ^8^Department of Emergency Medicine, Chang Gung Memorial Hospital, Keelung, Taiwan; ^9^Department of Medical Imaging and Intervention, Chang Gung Memorial Hospital, Linkou, Taoyuan, Taiwan; ^10^Department of Diagnostic Radiology, Chang Gung Memorial Hospital, Keelung, Taiwan; ^11^Department of Medical Imaging and Radiological Sciences, Chang Gung University, Taoyuan, Taiwan; ^12^Medical Imaging Research Center, Institute for Radiological Research, Linkou Chang Gung Memorial Hospital, Chang Gung University, Taoyuan, Taiwan

**Keywords:** fixel-based analysis, Huntington’s Disease, diffusion-tensor imaging, magnetic resonance imaging, neurodegeneration

## Abstract

Microstructure damage in white matter might be linked to regional and global atrophy in Huntington’s Disease (HD). We hypothesize that degeneration of subcortical regions, including the basal ganglia, is associated with damage of white matter tracts linking these affected regions. We aim to use fixel-based analysis to identify microstructural changes in the white matter tracts. To further assess the associated gray matter damage, diffusion tensor-derived indices were measured from regions of interest located in the basal ganglia. Diffusion weighted images were acquired from 12 patients with HD and 12 healthy unrelated controls using a 3 Tesla scanner. Reductions in fixel-derived metrics occurs in major white matter tracts, noticeably in corpus callosum, internal capsule, and the corticospinal tract, which were closely co-localized with the regions of increased diffusivity in basal ganglia. These changes in diffusion can be attributed to potential axonal degeneration. Fixel-based analysis is effective in studying white matter tractography and fiber changes in HD.

## Introduction

Huntington’s Disease (HD) is an autosomal dominant genetic disease that results in progressive neurodegeneration. It is caused by the expansion of cytosine-adenine-guanine (CAG) trinucleotide repeats in the huntingtin (*HTT*) gene on chromosome 4, which is responsible for the expression of the protein huntingtin (Nance, 2017). Symptoms are typically exhibited between the ages of 35 and 40, and include deterioration of motor skills, with chorea parkinsonism, cognitive decline, and psychiatric changes, comprising depression and apathy (Ross et al., 2014). Diagnosis is based on genetic testing and symptoms are evaluated using clinical rating scales such as the Unified Huntington’s Disease Rating Scale (UHDRS; Huntington Study Group, 1996).

Magnetic resonance imaging (MRI) is a non-invasive technique that provides insight about diagnosis and treatment for neurological diseases. Structural MRI revealed extensive gray and white matter atrophy in the brains of patients with HD (Hobbs et al., 2010). Noticeably, caudate nuclei atrophy has been observed even before the onset of HD symptoms, with atrophy rates directly related to CAG repeat count (Hobbs et al., 2010). Positron emission tomography (PET) studies also reported hypometabolism in the striatum and the cortex of both pre-/symptomatic patients with HD (Ciarmiello et al., 2006), suggesting that activity and function of these regions could be affected. Large-scale longitudinal MRI cohort studies focused mainly on identifying biomarkers of HD that can indicate disease progression before and after clinical diagnosis throughout different stages (Paulsen et al., 2008; Tabrizi et al., 2012; Domínguez D et al., 2013). Both PET and functional MRI have been used to evaluate brain functions during HD progression (Paulsen, 2009).

Diffusion MRI is often used as a non-invasive method of observing microstructural and macrostructural alterations in gray and white matter over time. The use of diffusion MRI has become widespread in white matter analysis due to its sensitivity to microstructural tissue properties. Analysis of changes in water diffusivity in patients with HD might provide insight on the damage that the brain might undergo as the disease progresses. The subcortical nuclei in the basal ganglia have well-interconnected white matter projections (Cacciola et al., 2017), which partly explain the reported association of these structures with motor control and learning, as well as executive function, emotion, and behavior (Lanciego et al., 2012). In HD, the severity of motor impairments is often found to be correlated to volume loss in the basal ganglia, particularly in the nucleus accumbens, caudate nucleus, putamen, and globus pallidus (Coppen et al., 2018). Widespread white matter neurodegeneration has been observed in longitudinal studies of patients with HD, especially in the corpus callosum and the internal capsule (Rosas et al., 2006). The cingulum and striatal projection might undergo changes in microstructure during disease progression (Poudel et al., 2015). We therefore hypothesize that the atrophy that takes place in subcortical regions of patients with HD, in particular the basal ganglia, is associated with the damage of white matter tracts linking the affected subcortical regions.

Analysis in most MRI studies today employ voxel-based morphometry, for example, the large scale Predict-HD (Paulsen et al., 2008), TRACK-HD (Tabrizi et al., 2012), and IMAGE-HD studies (Domínguez D et al., 2013). Fixel-based analysis is a novel model for analysis of diffusion MRI which represents fibers within voxels (Raffelt et al., 2017). It consists of spatial normalization, specifically by estimating the fiber orientation distribution (FOD) of diffusion MRI, and registering the FOD images to an unbiased study-specific template through a non-linear warp. Fiber density (FD) and fiber bundle cross-section (FC) are obtained, respectively. FD undergoes modulation with FC to give fiber density and cross-section (FDC; Raffelt et al., 2017). FD represents changes in the volume of restricted water within a voxel, while FC represents changes in the number of voxels occupied by a fiber bundle, and FDC is a combined measure of FDC (Raffelt et al., 2017). A combination of connectivity based fixel enhancement (CFE) and connectivity based smoothing adjusts the test statistics of fixels and allows for representation of fiber tracts (Raffelt et al., 2015).

To investigate changes that could arise from the onset and progression of HD, we conducted a study on patients with HD and healthy control subjects. The specific aims of this study were to investigate possible white matter damage that could be linked to motor and mental deterioration in patients with HD. Secondly we proposed to identify the subcortical damage that could be affected during the course of HD.

## Materials and Methods

The cross-sectional study was approved by the Institutional Review Board of Chang Gung Medical Foundation (201301056B0, 201507105B0) in Linkou, Taiwan, and complied with the Declaration of Helsinki. All participants gave written informed consent before participating in the study.

### Participants

Sixteen patients with HD were recruited from neurology clinics in a tertiary referential medical center (ChangGung Memorial Hospital, Linkou). Additional 16 healthy control participants were recruited from the local community.

Patients were included if the diagnosis from a neurological examination was confirmed by genetic assessment of CAG expansion in the *HTT* gene. The exclusion criteria are (1) significant major systemic disease, such as renal failure, heart failure, stroke, acute myocardial infarction, unstable angina, poorly controlled diabetes mellitus, and poorly controlled hypertension; (2) pregnant or breast feeding women; (3) severe dementia; (4) any documented abnormality of the brain caused by etiologies other than HD in MRI or ^18^FDG PET studies, except for mild cortical atrophy; (5) history of intracranial operation, including thalamotomy, pallidotomy, and/or deep brain stimulation; and (6) significant physical disorders or neuropsychiatric disorders. Subjects with contraindications to MR scanning (e.g., metal implants, cardiac pacemaker, or implantable defibrillator) were excluded from the study.

After being screened for inclusion and exclusion criteria, four patients were determined to be pre-symptomatic, who were not included in the final analysis due to the small sample number. The 12 symptomatic patients (Male/Female = 7/5, aged 50.1 ± 9.53 years) and 12 healthy control participants (Male/Female = 5/7, aged 50.1 ± 9.69 years) underwent MRI, cognitive, physical, and neurological examinations. The 12 patients were from 11 separate families, with one pair of siblings. The patients’ assessments included the cognitive status [evaluated by Mini-Mental State Examination (MMSE)], the clinical severity (evaluated using the UHDRS) and the CAG score (the number of CAG repeats present in the *HTT* gene). Specifically, the motor, independence, and functional scales of each patient with HD were measured. The motor scale reflects the severity of motor symptoms such as abnormal eye movements, chorea, dystonia, difficulty in speech, impaired rapid and alternating hand movements, gait disturbance, bradykinesia or rigidity, and loss of postural stability. The independence scale evaluates the extent of external help patients need in order to care for themselves, and the functional scale assesses if the symptom has affected patients’ abilities to carry out tasks, such as for their occupation, handling their finances, or carrying out domestic chores (Huntington Study Group, 1996). The demographic data of the study’s participants is shown in Table 1.

**TABLE 1:** Demographic description between patients with Huntington’s Disease (HD) and healthy control.

No.	Age	Sex	CAG No.^*a*^	UHDRS^*b*^			MMSE^*c*^
				
				Motor	Independence	Functional	
1	36	F	49	76	50	5	21
2	60/58	M	43	52	60	4	17
3	52	F	42	52	50	1	22
4	35	M	46	47	50	3	25
5	46/45	F	45	39	70	7	28
6	46/47	F	43	58	50	1	22
7	54	M	42	50	50	3	25
8	58	M/F	44	9	100	12	26
9	66/68	F/M	42	39	70	6	19
10	53	M/F	43	13	100	13	30
11	54	M	43	17	100	11	26
12	41	M/F	48	22	80	8	22

*Each row represents a pair of patient with HD and healthy control as closely matched as possible. If different, age and sex are displayed as the patient’s/the healthy control’s, respectively.*

*^*a*^The CAG score refers to the number of cytosine-adenine-guanine repeats present in the huntingtin (*HTT*) gene for each patient with HD.*

*^*b*^The Unified HD Rating Scale.*

*^*c*^Mini-Mental State Examination (MMSE) scores of the HD patients are shown.*

### Imaging Procedure

Images were acquired from 3T MR scanner (Trio, Magnetom, Siemens, Erlangen, Germany), including T_1_-weighted magnetization-prepared rapid acquisition gradient echo (T1-MPRAGE) and diffusion weighted images. In order to minimize the patient excessive motion, a fixation pad was used. The total acquisition time was 20 min 10 s.

The imaging parameters for T_1_-weighted images were as follows: repetition time (TR)/echo time (TE) = 1,700/2.63 ms, number of slices = 160, voxel size = 1 mm × 1 mm × 1 mm, inversion time = 900 ms, flip angle = 9°, matrix size = 224 × 256, field of view (FOV) = 224 mm × 256 mm.

Diffusion weighted images were acquired using a spin-echo echo planar imaging (EPI) sequence with the following parameters: TR/TE = 5,200/92 ms, voxel size = 2 mm × 2 mm × 3 mm, matrix size = 64 × 64, number of slices = 40 and *b*-value = 0 and 1,000 s/mm^2^. The diffusion weighted gradients were applied along 12 non-collinear directions.

### Image Analysis

Fixel-based analysis was carried out using single-tissue constrained spherical deconvolution in MRtrix3 according to its recommended protocol (Raffelt et al., 2017). In short, the diffusion weighted images were denoised using Marchenko–Pastur principal component analysis (Veraart et al., 2016; Tournier et al., 2019). Gibbs ringing was removed based on local subvoxel-shifts (Kellner et al., 2016; Tournier et al., 2019), and correction was conducted for motion, distortion and bias field (Smith et al., 2004; Andersson and Sotiropoulos, 2016). The diffusion weighted images were upsampled to an isotropic voxel size of 1.3 mm using cubic interpolation. Normalization was performed to a group template by using non-linear co-registration. The white matter measures of FD, FC, and FDC were calculated for each voxel.

Subcortical gray matter segmentation was performed using freeware FMRIB Software Library (Jenkinson et al., 2012), including co-registration of T_1_-weighted images and diffusion-weighted images, normalized to the Montreal Neurological Institute-152 template after noise reduction (Fonov et al., 2011). Regions of interest were selected bilaterally, comprising the caudate nuclei, putamina, pallidi, hippocampi, and thalami. The mean values of the tensor-derived indices were recorded from each region of interest.

### Statistical Analysis

Statistical tests were conducted using IBM SPSS Statistics 24 (SPSS Inc., Chicago, IL, United States) (IBM Corp, 2016). Statistical analysis in fixel-derived metrics, including FD, FC, and FDC, was performed using MRtrix3.

In demographic data, Mann–Whitney U test was used to compare age differences between patient and healthy control populations, while a chi-square test was used to compare differences in sex. In analysis of tensor-derived indices of subcortical gray matter, Mann–Whitney U test was used to compare differences between patients and healthy controls. The correlation between the tensor-derived indices and the patients’ assessment was evaluated using Spearman’s rank correlation coefficient. In all tests, significance was reached at a threshold of *p* < 0.05, after correction for multiple comparisons using Bonferroni correction if necessary.

For fixel-based analysis, non-parametric permutation testing, CFE (Raffelt et al., 2015), and general linear model, were used to identify differences between groups in each fixel-derived metric. Linear regression was performed between fixel-derived metrics and clinical assessment scores. In both analyses, sex and age were set as covariates. The family-wise error corrected *p* < 0.05 was used to determine statistical significance.

## Results

### Fixel-Based Analysis

**FIGURE 1:**
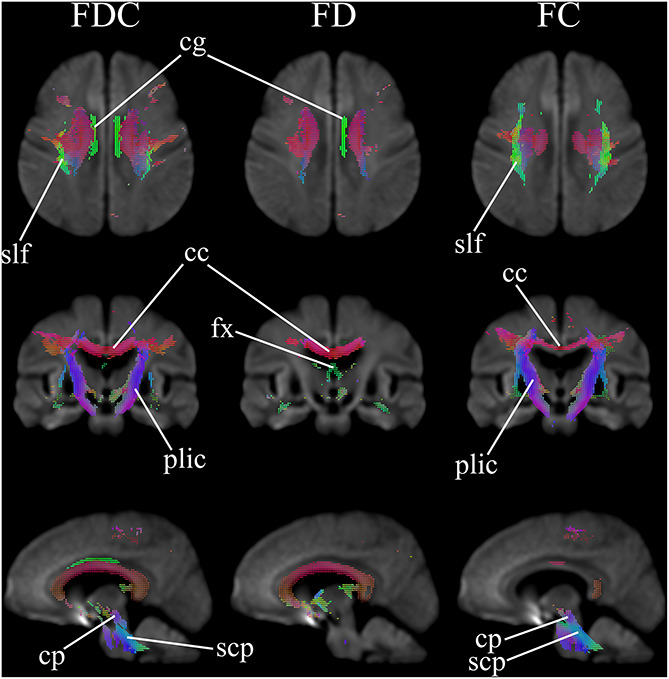
Areas of significant reductions in fiber density and cross-section (FDC), fiber density (FD), and fiber bundle cross-section (FC) in patients with Huntington’s Disease compared to healthy control participants, displayed in the axial, coronal, and sagittal views. Significant fixels (family-wise error corrected *p* < 0.05) are illustrated and colored according to fiber direction. FDC, fiber density and cross-section; FD, fiber density; FC, fiber bundle cross-section; cg, cingulum; slf, superior longitudinal fasciculus; cc, corpus callosum (forceps minor, major, and corona radiata white matter fibers); fx, fornix (pre- and post-commissural fibers); plic, posterior limb of internal capsule (corticospinal fibers and middle thalamic radiation); cp, cerebral peduncles (extrapyramidal tracts); scp, superior cerebellar peduncles (extrapyramidal tracts).

Figure 1 comparatively shows difference in fixel-derived metrics from patients with HD and healthy control participants along three different views. Patients showed reduced fixels located in widespread white matter, noticeably in the forceps minor, major, corona radiata, corpus callosum, the corticospinal tract, middle thalamic radiation, and the posterior limb of the internal capsule (Figure 1, panel 1 and 3 for leftmost and rightmost columns). Besides corpus callosum and internal capsule, FDC and FC reductions were observed in the superior longitudinal fasciculus and the extrapyramidal tracts in the cerebral peduncles as well as in the superior cerebellar peduncles, with reduced FDC seen in the cingulum. Apparent FD reductions were observed in the cingulum and the pre- and post-commissural fibers in the fornix.

**FIGURE 2:**
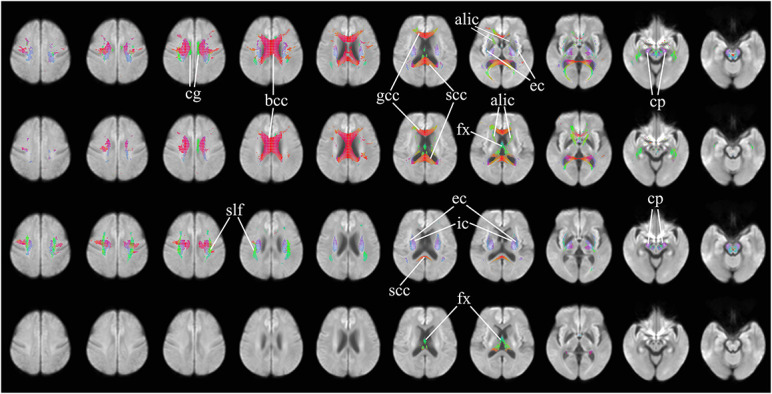
Areas of significant differences in FDC, FD, and FC in patients with HD compared to healthy control participants, displayed in the axial view. Significant fixels (family-wise error corrected *p* < 0.05) are illustrated and colored according to fiber direction. Top row: FDC reductions in patients with HD compared to healthy controls; second row: FD reductions in patients with HD compared to healthy controls; third row: FC reductions in patients with HD compared to healthy controls, areas of higher FC in patients with HD compared to healthy controls; bottom row: areas of higher FC in patients with HD compared to healthy controls. cg, cingulum; bcc, body of corpus callosum (corona radiata white matter fibers); gcc, genu of corpus callosum (forceps minor fibers); scc, splenium of corpus callosum (forceps major fibers); alic, anterior limb of internal capsule (anterior thalamic radiation and frontopontine fibers); ec, external capsule (claustrocortical fibers, uncinate fasciculus, and inferior frontal occipital fasciculus); cp, cerebral peduncles (extrapyramidal tracts); fx, fornix (pre- and post-commissural fibers); slf, superior longitudinal fasciculus; ic, internal capsule (white matter projection fibers of the corona radiata).

Figure 2 further highlights the significant changes, as seen in the axial view, of fixel-derived metrics in patients. FDC reductions (Figure 2, top row) can be noticed bilaterally in the genu, body, and splenium of the corpus callosum, and in the anterior limb of the internal capsule, consistent with the regions of reduced apparent FD (Figure 2, second row). Additional region with reductions in apparent FD was observed in the fornix and in areas surrounding the lateral ventricles. In addition, FDC reductions were noticed in the external capsule, the primary motor cortex, premotor cortex, the cerebral peduncles, and the cingulum (Figure 2, top row).

**FIGURE 3:**
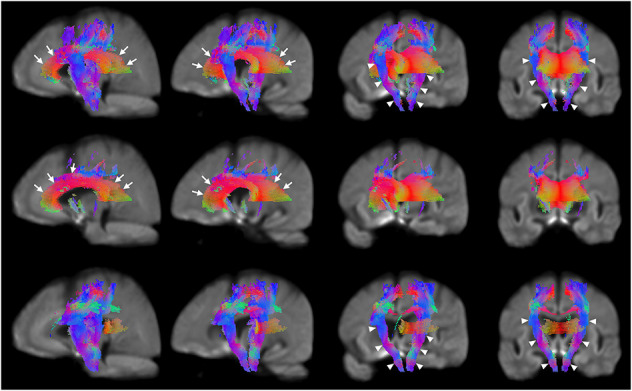
Areas of significant reductions in FDC, FD, and FC in patients with HD compared to healthy control participants, displayed as a rotation from sagittal to coronal views. Significant fixels (family-wise error corrected *p* < 0.05) were illustrated and colored according to fiber direction. The corpus callosum (seen in the leftmost and second columns) and internal capsule (seen in the third and rightmost columns) were labeled using white arrows. Top row: FDC reductions in corticospinal tract of patients with HD compared to healthy controls with rotation from sagittal to coronal views; second row: FD reductions in corticospinal tract of patients with HD compared to healthy controls with rotation from sagittal to coronal views; bottom row; FC reductions in corticospinal tract of patients with HD compared to healthy controls with rotation from sagittal to coronal views.

Significant reductions in FC (Figure 2, third row) are observed in the neighborhood of the basal ganglia, the internal capsule, the external capsule, the splenium of the corpus callosum and in the bilateral cerebral peduncles. Reductions in the primary motor cortex and premotor cortex are also observed. Increased FC was observed in the fornix of patients as compared to healthy control participants (Figure 2, fourth row). In addition, reduced FDC and FC were observed in the corticospinal tract (Figure 3). Animations further illustrating this are included in the Supplementary Videos 1–3.

### Diffusion Tensor Analysis

**FIGURE 4:**
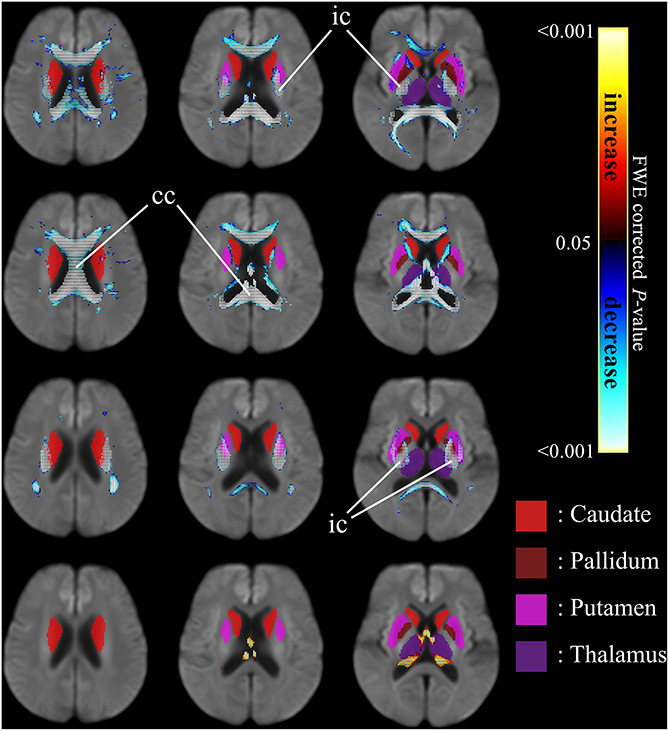
Significant differences in tensor-derived indices from subcortical gray matter segmentation (shown in solid colors) and significant reductions in fixel-derived metrics in surrounding neighborhood regions, between patients with HD and healthy control participants. Areas of significant differences in FDC, FD, and FC displayed in the axial view. Significant fixels (family-wise error corrected *p* < 0.05) were illustrated and colored according to *p*-values. Cold colors denote reduction, whereas warm colors denote increase. Top row: FDC reductions in patients with HD compared to healthy controls; second row: FD reductions in patients with HD compared to healthy controls; third row: FC reductions in patients with HD compared to healthy controls; bottom row: areas of higher FC in patients with HD compared to healthy controls. ic, internal capsule; cc, corpus callosum.

Figure 4 shows the co-localization of significant differences in FDC, FC, and FD of white matter fibers and tensor-derived indices of subcortical gray matter regions (displayed in green), displayed in the axial view. It can be observed that the affected white matter tracts, such as the internal capsule and corpus callosum, are closely positioned to these subcortical regions of interest.

Table 2 shows the tensor-derived indices in patients with HD and in healthy controls from the 10 selected subcortical regions of interest, including the regions shown in Figure 4. Significantly higher Axial Diffusivity can be found in all regions, i.e., the bilateral caudate nuclei, globus pallidi, hippocampi, putamina, and thalami. Increase in Mean Diffusivity (MD) were also found in all regions except the right globus pallidus. Increase in Radial Diffusivity (RD) were located in most regions of investigation, except the globus pallidi and right putamen. However, an increase in Fractional Anisotropy (FA) was only noticed in the right caudate nucleus.

**TABLE 2:** Tensor derived indices in patients with HD and healthy control participants.

	AD		RD		MD		FA	
				
Regions	HD	HC	HD	HC	HD	HC	HD	HC
LCaud	1.63 ± 0.26**	1.15 ± 0.14	0.99 ± 0.21**	0.72 ± 0.10	1.20 ± 0.23**	0.87 ± 0.11	0.38 ± 0.04	0.34 ± 0.03
RCaud	1.64 ± 0.18**	1.21 ± 0.15	1.04 ± 0.17*	0.79 ± 0.11	1.24 ± 0.17**	0.93 ± 0.12	0.37 ± 0.05*	0.31 ± 0.03
LGPal	1.29 ± 0.09**	1.06 ± 0.05	0.63 ± 0.13	0.51 ± 0.05	0.85 ± 0.11*	0.69 ± 0.04	0.50 ± 0.09	0.49 ± 0.05
RGPal	1.29 ± 0.10*	1.02 ± 0.05	0.55 ± 0.14	0.46 ± 0.06	0.77 ± 0.14	0.64 ± 0.05	0.54 ± 0.09	0.54 ± 0.06
LHipp	1.29 ± 0.11**	1.20 ± 0.11	0.94 ± 0.09**	0.76 ± 0.08	1.10 ± 0.09**	0.91 ± 0.09	0.31 ± 0.05	0.33 ± 0.03
RHipp	1.29 ± 0.12**	1.23 ± 0.08	0.97 ± 0.08**	0.77 ± 0.06	1.14 ± 0.09**	0.92 ± 0.07	0.31 ± 0.04	0.33 ± 0.02
LPuta	1.29 ± 0.13**	1.02 ± 0.04	0.65 ± 0.07**	0.56 ± 0.04	0.84 ± 0.06**	0.71 ± 0.03	0.42 ± 0.05	0.40 ± 0.04
RPuta	1.29 ± 0.14**	0.98 ± 0.04	0.61 ± 0.08	0.52 ± 0.03	0.81 ± 0.07**	0.68 ± 0.03	0.46 ± 0.06	0.42 ± 0.04
LThal	1.29 ± 0.15**	1.20 ± 0.08	0.79 ± 0.07**	0.64 ± 0.04	1.00 ± 0.08**	0.82 ± 0.05	0.45 ± 0.03	0.44 ± 0.03
RThal	1.29 ± 0.16**	1.19 ± 0.07	0.75 ± 0.08**	0.63 ± 0.04	0.97 ± 0.08**	0.81 ± 0.05	0.47 ± 0.04	0.45 ± 0.04

*Data are presented as means ± standard deviations. ^∗^*p* < 0.005; ^∗∗^*p* < 0.001. AD, axial diffusivity; RD, radial diffusivity; FA, fractional anisotropy; MD, mean diffusivity; HD, Huntington’s Disease; HC, healthy control; L, left; R, right; Caud, caudate; GPal, globus pallidi; Hipp, hippocampi; Puta, putamina; Thal, thalami.*

### Correlations Between Diffusivity and Clinical Assessment Scores

The regions with significant correlations between tensor-derived indices and the clinical assessment scores are listed in Table 3. Diffusivities (axial/radial/mean) in the left caudate can be correlated with the motor, independence, and functional scores of the UHDRS. In the left thalamus, significant correlations can be observed with motor and functional scores.

**TABLE 3:** Significant correlations between tensor-derived indices in subcortical brain regions and clinical scores in patients with HD.

	AD	RD	MD	FA
Motor				
Left caudate	0.828 (0.001)	0.947 (0.000)	0.933 (0.000)	n.s.
Left thalamus	0.754 (0.003)	0.877 (0.000)	0.842 (0.001)	n.s.
Independence				
Left caudate	−0.794 (0.002)	−0.882 (0.000)	−0.886 (0.000)	n.s.
Functional				
Left caudate	−0.853 (0.000)	−0.874 (0.000)	−0.881 (0.000)	n.s.
Left thalamus	−0.758 (0.004)	n.s.	n.s.	n.s.

*Data are expressed as Spearman’s Rank Correlation Coefficient (*p*-value) for each subcortical region. AD, axial diffusivity; RD, radial diffusivity; FA, fractional anisotropy; MD, mean diffusivity. n.s, not significant.*

No correlation was found between fixel-derived metrics and the clinical assessment scores under investigation, namely the motor, independence, and functional scores of the UHDRS, MMSE, and number of CAG repeats.

## Discussion

This study sought to investigate white matter microstructural differences in patients with HD using fixel-based analysis. Compared to the healthy control participants, the main findings in patients include: (1) between-group differences in fixel-derived indices, noticeably located bilaterally in the corpus callosum and internal capsule, (2) reduced FDC and FC along the corticospinal tract, and (3) increased FC in the fornix, despite reduction in FDC and apparent FD.

Fixel-based analysis addresses two limitations of voxel-based analysis in studying white matter microstructure: (1) multiple fiber populations within voxels – 90% of white matter voxels are believed to contain multiple fibers, and (2) understanding the axonal changes along an entire fiber tract spanning multiple voxels (Raffelt et al., 2015). These problems stem in part from voxel-based analysis conducting smoothing using a uniform Gaussian isotropic kernel, which has been found to lead to reduced sensitivity and specificity of pathology detection particularly for white matter (Van Hecke et al., 2010). In contrast, the FOD estimation and fixel reorientation steps in fixel-based analysis allow multiple fiber populations in each voxel to be resolved. The reductions in fixel-derived metrics might suggest that the potential atrophy observed in patients with HD could be associated with axonal degeneration. The affected regions, including the corpus callosum and the internal capsule, are in line with those in morphometry studies (Hobbs et al., 2010). The potential axonal degeneration in these regions and other major tracts such as the superior longitudinal fasciculus and uncinate fasciculus might explain why HD affects multiple regions of the brain with such widespread effects on motor and cognitive functions.

Our results suggest that fixel-based analysis may be a sensitive tool in the identification of white matter damage in HD. The fixel-derived metrics show the potential to provide new insight about informational relay within the fiber bundles (Raffelt et al., 2017). A recent investigation on presymptomatic and very early stage HD demonstrated similar white matter alterations in the corpus callosum and corticospinal tract (Adanyeguh et al., 2021). Our study might provide additional image-based evidence that axonal degeneration is likely to occur over the manifestation of HD.

### Role of Corpus Callosum, Internal Capsule, and Corticospinal Tract

At prodromal disease stages, microstructural impairments have been identified for major white matter bundles including the corpus callosum, the anterior thalamic radiation, and the corticospinal tract (Novak et al., 2014; Phillips et al., 2014; Matsui et al., 2015; Odish et al., 2015; Poudel et al., 2015; Steventon et al., 2016), which might possibly support the observation of white matter loss during early disease onset, as was followed by gray matter degeneration (Fischer et al., 2004; Gatto et al., 2015). Our study found a reduction in fixel-derived metrics in the corpus callosum of patients with HD compared to healthy control participants. The corpus callosum is the largest white matter tract in the brain and is involved in the transfer of information between both hemispheres (Aboitiz et al., 1992). The fibers in these areas are responsible for the transfer of motor, somatosensory, auditory and visual information, as well as higher cognitive functions (Goldstein and Mesfin, 2019). Degeneration or damage in the corpus callosum can be linked with deficits in attention, working memory and motor impairments (Phillips et al., 2013). The corpus callosum is among the first structures to exhibit volume loss, along with the striatum and posterior white matter tract (Tabrizi et al., 2012) and can be regarded as the white matter structure which displays the most evident changes during disease onset (Ross et al., 2014), even if no associated changes in cognitive performance (Crawford et al., 2013). The reduction of fixel-derived metrics in the corpus callosum in our study might be associated with the motor and cognitive symptoms observed in patients with HD, perhaps due to hindrance of interhemispheric communication.

Reduction of both FDC and FD were noticed in the anterior limb of the internal capsule. The anterior limb of the internal capsule plays a critical role in information relay in the brain (Emos and Agarwal, 2019), which might be related to the cognitive and motor symptoms observed in HD. Abnormalities in the anterior limb have been linked to anhedonia in major depressive disorder and schizophrenia (Mithani et al., 2020). In HD, the rate of atrophy in the internal capsule is known to increase with CAG repeat length, and associated with increased UHDRS motor scores (Hobbs et al., 2010). Changes in FA in the anterior limb of the internal capsule were also found to be correlated with poorer cognitive performance (Sweidan et al., 2020). Alterations in the internal capsule in our study might likely play a role in the manifestation of HD and the severity of cognitive symptoms.

Damage in the corticospinal tract was observed, as reflected by reduction in both FDC and FC. Dysregulated conductivity in the corticospinal tract has been hypothesized to occur in HD using electrophysiological studies (Georgiou-Karistianis et al., 2004). The corticospinal tract is essential for movement control (Welniarz et al., 2017). Change of fixel derived indices in the corticospinal tract may account for the presentation of motor impairments in HD, in particular voluntary movement. We demonstrated through fixel-based analysis that corticospinal tract degeneration might likely occur in the disease course.

### Alterations of Tensor-Derived Indices in Basal Ganglia

Our study reported white matter alterations in the neighborhood of basal ganglia by using fixel-based analysis. The co-localization of changes in diffusivities in the basal ganglia with alterations in fixel-derived indices in the neighboring white matter might suggest potential connection between the microstructural white matter changes with the anomalies in gray matter regions. The axonal damage in the major white matter tracts as reflected by fixels could be closely associated with the functions of the basal ganglia. For instance, reduced FDC was observed in the internal capsule, which separates the caudate nucleus and thalamus from the lentiform nucleus. The internal capsule contains fibers which are functionally related to somatosensory relay involving the thalamus (Emos and Agarwal, 2019). The basal ganglia and corticospinal tract are functionally and anatomically related in the control of voluntary movement (Phillips et al., 2015), further suggesting that damage or degeneration observed in these regions are related to motor symptoms in HD.

Significant increases in diffusivity (axial/radial/mean) were noticed in the basal ganglia and thalamus in patients with HD compared with healthy control participants, indicating increased free diffusion and thus tissue damage. Atrophy in the basal ganglia was previously reported between patients with HD and their first-degree relatives at risk of developing the disease (Valdés Hernández et al., 2019). In this study, we also noticed significant correlation between tensor-derived indices and UHDRS scores, particularly in the left caudate nucleus and left thalamus (Table 3). The observation of basal ganglia degeneration and damage in neighboring white matter could be associated with motor dysfunction in HD. Diminished motor coordination in HD is often attributed to basal ganglia neuronal loss, particularly in the striatum (Coppen et al., 2018). Impaired corticostriatal connectivity has been implicated in the development of ideomotor limb apraxia in HD (Hamilton et al., 2003). Increased diffusivity in the basal ganglia together with white matter neurodegeneration in our study could provide an explanation for the pathophysiology of symptoms in HD. The observed co-localization between subcortical damage and white matter tract alterations might suggest a close relationship between both during the course of HD which would be worth further investigation.

Caudate atrophy is usually presented in patients with HD and has been suggested to be a biomarker indicating disease progression in pre-symptomatic and symptomatic individuals (Tabrizi et al., 2012). Changes in tensor-derived indices located in the right caudate nucleus in our study was in line with the reverse changes as reported by Gatto and Weissmann (2019). This might be potentially attributed to the surrounding white matter loss which caused the appearance of comparatively more organized striatal gray matter (Douaud et al., 2009; Singh et al., 2013). Studies on preclinical mouse models have indicated that cortical neuronal death is not proportionally accompanied by white matter tract degeneration (Rattray et al., 2013), and early white matter changes are a main feature of neuronal and axonal connectivity impairment in fluorescent-tagged animals (Gatto et al., 2015). Further studies tracking gray and white matter degeneration in both presymptomatic and symptomatic HD populations might help to elucidate the relationship between microstructural white matter changes and anomalies in gray matter regions.

### Additional Regions of Interest

Fixel based analysis showed reduced FDC and FD together with an increased FC in the fornix. Studying the tractography and diffusivity of the fornix has been challenging due to complications in the diverging fiber populations and the curved morphology (Thomas et al., 2011). Our study might provide new insight of the microenvironment changes in these regions with complicated fiber structures. Neuronal degeneration in the fornix has been identified in schizophrenia, Alzheimer’s Disease and multiple sclerosis (Thomas et al., 2011), as is often associated with a loss in long-term memories and impairment of new memory formation (Thomas et al., 2011). Our finding of changes in the fornix might provide motivation to investigate memory impairment associated with HD by including more specific examination such as the Montreal Cognitive Assessment (Mestre et al., 2018).

Reductions in FDC and FC were seen in the superior cerebellar peduncles, suggesting fiber damage in this white matter tract. The superior cerebellar peduncles might play a role in the link between the dentate nucleus, internal globus pallidus and substantia nigra (Milardi et al., 2016), which could potentially allow for modulatory effects between the dentate nucleus and the basal ganglia (Milardi et al., 2019). The cerebral peduncles are involved in the transmission of motor signals from the brain down the spinal cord through the corticospinal tract (Watson and Harvey, 2009). Injury or dysfunction of the superior cerebellar peduncles have been linked to other motor disorders including Friedreich’s ataxia (Della Nave et al., 2011) and dentatorubro-pallidoluysian atrophy, which, like HD, is characterized by expanded CAG repeats (Taoka et al., 2007). In the cerebral peduncles, reduced FDC and FC might suggest a reduced information relay. Degeneration of the cerebral peduncles, in particular through Wallerian degeneration or *trans-*synaptic degeneration, can be observed in events of motor pathway infarcts such as stroke, and is correlated with corticospinal tract injury (Mark et al., 2008). Damage in the cerebral peduncles in our study therefore might suggest a role in the appearance of particular motor symptoms in HD, for example, gait impairment.

### Clinical Implications and Limitations of Fixel-Based Analysis

HD severity is often attributed to basal ganglia degeneration. Fixel-based analysis reveals microstructural and macrostructural alterations of white matter that could be present within specific fiber tracts. Through fixel-based analysis, this study shows that HD could be characterized by widespread white matter loss in major fiber structures, including the corpus callosum, internal capsule, and the corticospinal tract. This might provide additional information on degeneration in white matter tracts when compared to conventional voxel-based analysis. It may be especially helpful in identifying and tracking white matter alterations in individuals at risk as it is believed that white matter degeneration occurs progressively leading up to disease onset (Rosas et al., 2006). Additional studies comparing clinical scores of patients with HD over time using fixel-based analysis might further illustrate the extent of motor and cognitive decline in patients. Along with correlation with clinical scores, fixel-based analysis could also be conducted to provide new insight into how neurodegeneration influences the severity of motor and cognitive symptoms as the disease progresses and provide data on relevant stages for clinical intervention and treatment before HD onset.

The metrics from fixel-based analysis, specifically the estimation of FD, have been shown to exhibit a degree of dependence on *b*-values, especially in association tracts (Genc et al., 2020). Further investigations could employ improved quality of diffusion images collected with High Angular Resolution Diffusion Imaging (Tuch et al., 2002) in multi-shells and increased *b*-values, or novel analysis methods such as non-gaussian methods, including neurite orientation dispersion and density imaging (NODDI; Zhang et al., 2018) and continuous time random walk (CTRW) models (Gatto et al., 2019). In addition, reverse-encoding image scans could be acquired or deep learning methods could be employed to correct for susceptibility induced artifacts (Schilling et al., 2020).

Given the nature and rarity of HD, sample sizes of investigations involving human patients tend to be small and limited to families. However, *post hoc* power analysis indicated that the sample size in this study is sufficient and appropriate. Despite the small population size, we observed significant differences in white matter using fixel-based analysis, which are in agreement with studies using voxel-based morphometry. Our study demonstrated that fixel-based analysis can identify regions of white matter degeneration in HD. Future investigations that involve increased number of pre-HD subjects could be useful in understanding white and gray matter degeneration over the course of HD onset.

## Conclusion

Reduced fixel-derived metrics in white matter and increased diffusivities in basal ganglia were observed in patients with HD, the co-localization of which might suggest potential connection between the white matter damage and the gray matter anomalies. Fixel-based analysis could provide new information on white matter tracts affected in patients of HD, and therefore could be potentially helpful in the development of new therapeutic interventions.

## Data Availability Statement

The raw data supporting the conclusions of this article will be made available by the authors upon reasonable request.

## Ethics Statement

The studies involving human participants were reviewed and approved by the Ethics Committee of the Chang Gung Medical Foundation Institutional Review Board (approval numbers: 201301056B0 and 201507105B0). The patients/participants provided their written informed consent to participate in this study.

## Author Contributions

SLO: conceptualization, methodology, writing – original draft, and writing – review and editing. C-MC and Y-RW: resources, investigation, and writing – review and editing. MV: methodology, software, statistical analysis, and writing – review and editing. C-CT: methodology and data curation. J-SC: formal analysis and writing – review and editing. Y-LC, Y-MW, and Y-CL: resources and investigation. J-JW: conceptualization, methodology, data curation, writing – review and editing, supervision, project administration, and funding acquisition. All authors contributed to the article and approved the submitted version.

## Conflict of Interest

The authors declare that the research was conducted in the absence of any commercial or financial relationships that could be construed as a potential conflict of interest.

## Publisher’s Note

All claims expressed in this article are solely those of the authors and do not necessarily represent those of their affiliated organizations, or those of the publisher, the editors and the reviewers. Any product that may be evaluated in this article, or claim that may be made by its manufacturer, is not guaranteed or endorsed by the publisher.

## Supplementary Material

Supplementary Video 1 A 3D visualization of the affected regions of the major fiber bundles in fiber density (FD) and cross-section (FDC).

Supplementary Video 2 A 3D visualization of the affected regions of the major fiber bundles in FD.

Supplementary Video 3 A 3D visualization of the affected regions of the major fiber bundles in fiber bundle cross-section (FC).
